# Novel *SLC6A9* variants in an adult presenting with GlyT1 encephalopathy

**DOI:** 10.1038/s10038-026-01497-4

**Published:** 2026-09-11

**Authors:** Mark Mencias, Meneka Kanagaratnam, Emma Matthews

**Affiliations:** Atkinson-Morley Neuromuscular Centre, Department of Neurology, St George’s University Hospitals NHS Foundation Trust, and Department of Psychology and Neuroscience, St George’s School of Health and Medical Sciences, City St George’s, University of London Cranmer Terrace, London, UK

**Keywords:** Genetic testing, Genetics research

## Abstract

Only 14 individuals have been thus far described with GlyT1 encephalopathy due to biallelic variants in the *SLC6A9* gene and the phenotypic picture is incomplete. Early mortality has been high, with only two known to survive into infancy. We report a 27-year-old individual who, to our knowledge, is the eldest person described with this ultra-rare disorder and who brings new phenotypic insights alongside potential prognosis for younger individuals. He is compound heterozygous for two novel *SLC6A9* variants. He shares many unifying clinical features, including neonatal hypotonia with later hypertonicity, respiratory failure, exaggerated startle and arthrogryposis. Clinical course stabilised after early childhood. Perampanel was of significant benefit for the exaggerated startle. Significant visual loss is unexplained. It may relate to the essential role of GlyT1 in retinal amacrine cells, although this mechanism remains speculative.

## Introduction

The *SLC6A9* gene codes for the sodium and chloride-dependent glycine transporter 1 (GlyT1). The main function of GlyT1 is to regulate the synaptic concentration of glycine, a neurotransmitter. GlyT1 encephalopathy is a relatively recently and rarely described autosomal recessive glycine encephalopathy due to variants in the *SLC6A9* gene [1, 2]. Also known as glycine encephalopathy with normal serum glycine, it is distinct from non-ketotic hyperglycinemia, which is another form of glycine encephalopathy due to deficient activity of the mitochondrial glycine cleavage system [3].

Only 14 individuals from 6 families with GlyT1 encephalopathy have been described and the phenotypic picture remains incomplete [1, 2, 4–6]. Early mortality has thus far been high, with only two individuals known to have survived into infancy [1, 2]. Common symptoms include neonatal hypotonia with later progression to limb hypertonicity, respiratory failure, dysmorphic features, arthrogryposis, startle-like responses provoked by loud sound and tactile stimulation with global developmental delay (Table 1).

**Table 1:** Summary of previously reported cases of *SLC6A9*-related GLYT1 encephalopathy

	Family 1	Family 2		Family 3			Family 4	
Consanguinity	Yes			Yes			Yes	
SLC6A9 variants	Homozygous c.1219 A > G p. (Ser407Gly)	Homozygous c.928_932delAAGTC p.(Lys310Phefs^a^31)		Homozygous c.1717 C > T (p.Gln573^a^)			Homozygous c.352 G > A p. (Val118Met)	
	Arab	Arab		Arab			NS	
Gender	Female	Female	Male	Female	Female	Male	Female	Male
Gestation	Term SVD	Pre-term 33 weeks CS foetal bradycardia	Termination at 13 weeks	Pre-term 35 weeks CS foetal distress	Term CS foetal distress	Pre-term 30 weeks CS	Termination (due to prenatal findings)	Termination (due to prenatal findings)
Age (at time of report)	15 months	2 years		Died at 18 days	Died at 2 days	Died at 7 months		
Ref	Alfadhel et al. [2]	Kurolap et al., [1]		Kurolap et al., [1]			Hauf et al., [4]	
Prenatal features	Polyhydramnios	Increased nuchal translucency, bilateral talipes, clenched hands, over-riding toes, polyhydramnios and dilation of the fourth ventricle	Hydrops	None at 17 weeks	Arthrogryposis	Bilateral talipes, hyperextension of knees	Increased nuchal translucency and severe arthrogryposis	Increased nuchal translucency and severe arthrogryposis
Neonatal hypotonia	yes	yes		yes	yes	yes		
Arthrogryposis	Yes	Yes		yes	yes	yes		
Later hypertonicity	None at 15 months	yes		yes	yes	yes		
Respiratory failure	yes	yes		yes	yes	yes		
Dysmorphic features	Microcephaly, broad forehead, esotropia, low-set ears, retrognathia, deep prominent philtrum	Trigonocephaly, long myopathic face, tent-shaped mouth, retrognathia, small upturned nose, depressed nasal bridge, long eyelashes, pronounced eyebrows, ptosis, low-set ears				Dolichocephaly, long myopathic face, tent-shaped mouth, small upturned nose, depressed nasal bridge, pronounced eyebrows, ptosis		
Post-natal Skeletal features		Talipes, hyperextension of knees, bilateral hip dislocation, contractures of elbows, wrists and hips, overriding toes, absent patellae		Left talipes, bilateral hip dislocation, flexion of elbows and knees, overriding fingers	Hyperextension of the knees, deformation of the hip joints	Talipes, hyperextension of knees, bilateral hip dislocation, contractures of hips and wrists, clenched fists		
Exaggerated startle or myoclonus	yes	yes		yes	yes	yes		
EEG	Normal	Mild generalised slowing						
MRI brain	Atrophy in the caudate nucleus, white matter changes	Lateral and third ventriculomegaly				Optic nerve atrophy, thin corpus callosum		

	Family 5		Family 6			Family 7	
Consanguinity	yes		yes			no	
SLC6A9 variants	Homozygous c.997delC p. (Arg333Alafs^a^3)		Homozygous c.250 G > A (p.Glu84Lys)^a^ (G2, G5 and father also heterozygous MTOR c.4444 C > T(p.Arg1482Cys)			Compound heterozygous c.1693del p.(Asp565fs) and c.1168_1170del p.(Phe390del)	
Ethnicity	Arab		Turkish	Turkish	Turkish	Turkish	Caucasian
Gender	Male	Female	Male (G1)	Female (G2)	Female (G5)	Male (G6)	Male
Gestation	Term	Spontaneous miscarriage at 16 weeks	32weeks CS due to foetal distress	32weeks CS due to hydrops	16 weeks (termination due to absent foetal heart activity)	32 weeks CS due to foetal bradycardia and hydrops	Term (breech delivery)
Age (at time of report)	Died at 13 days old		Died in the 5th hour	Died in 1st hour		Died in 1^st^ hour	27 years
Ref	Mademont-Soler et al. 2020		Dasar et al. 2022				This report
Prenatal features	Polyhydramnios, cryptorchidism, bilateral talipes and lack of lower limb movements	Increased nuchal translucency, bilateral talipes and reduced movement of the lower limbs	Bilateral talipes and hydrocephalus.	Polyhydramnios, knee hyperextension and talipes.	Bilateral talipes and bilateral flexion contractures of the elbows	Polyhydramnios and macrocephaly	Bilateral talipes
Neonatal hypotonia	Yes						Yes
Arthrogryposis	Yes						Yes
Later hypertonicity	Yes						Yes
Respiratory failure	Yes						Yes
Dysmorphic features	Hypertrichosis, increased skin on the back of the neck, retrognathia and long myopathic face			Hypertelorism, epicanthus inversus and laterally extending eyebrows		Hypertelorism, epicanthus inversus, laterally extending eyebrows and cleft palate	Long thin face, low set ears, high arched palate
Post-natal Skeletal features	Limitation of elbow and wrist extension, camptodactyly, dorsal scoliosis, rigid hyperflexed hips, talipes and digital contractures			Exam at birth: short neck, thoracic kyphosis, knee hyperextension and medially deviated toes		Exam at birth: joint contractures of hip, elbow and ankle, knee hyperextension, talipes, pes cavus, proximally placed halluces and bilateral overlapping fingers	Bilateral talipes, campylodactyly and arthrogryposis (hips, knees, shoulders) at birth. Later overlapping toes, pes cavus, kyphosis and prominent scapulae
Exaggerated startle	yes						yes
EEG	Burst suppression						Burst suppression
MRI brain							Normal

*CS* caesarean section

^a^Confirmed in G2, 5 and 6 only, as no sample available from G1

We report a 27-year-old male who is compound heterozygous for previously unreported variants in the *SLC6A9* gene. We believe him to be the eldest surviving person with GlyT1 encephalopathy, thus expanding the phenotypic spectrum and offering potential prognostic insight for younger individuals.

## Materials and methods

All clinical procedures were conducted as part of routine clinical care. Written informed consent was obtained to publish clinical details and photographs. DNA from the proband and their parents was submitted to an accredited NHS laboratory. The proband’s DNA underwent whole genome sequencing, and the results were confirmed by bi-directional Sanger sequencing.

## Results

### Clinical case

Talipes were noted on ultrasound during the first pregnancy of healthy Caucasian parents, which was otherwise uneventful. A male infant was born at term by breech delivery. Bilateral talipes, campylodactyly and arthrogryposis (hips, knees, shoulders) were noted at birth. He had hyper-reflexia and jittery movements and required ventilation days 1-5 post-natal. His EEG in the neonatal period had a pattern of burst suppression (Table 1). A single creatine kinase level was elevated at 3000IU/L. This prompted muscle biopsy, which was almost normal, a mild lipid increase attributed as most likely due to hypoxia. In retrospect, the elevated CK was post-IM injection and probably erroneous. Two CSF examinations in the neonatal period demonstrated elevated CSF glycine with a CSF to plasma glycine ratio of 0.08 and 0.098.

He was visually impaired, although the exact cause of visual loss was unclear, and had multiple surgeries for strabismus. Motor milestones were delayed and he initially used a walker and wheelchair. There was a gradual improvement in mobility and he was able to walk independently from age 5. Speech and language development was delayed, although progressively improved. In early childhood, he experienced dysphagia. He underwent surgery for orthodontic abnormalities. He also underwent multiple surgeries for contractures and had several admissions to the ICU with lower respiratory tract infection. He went on to attend mainstream school with educational support. When referred to us at age 26, he carried a diagnostic label of congenital myopathy.

He has completed a sports rehabilitation course at college. He walks independently at home and uses a stick outside. There are some trips and falls. He can navigate stairs but sometimes does this sitting down. He continues to have limited visual acuity. Dysphagia is much improved and requires only minimal modifications to the diet. He still experiences an exaggerated startle response to loud sounds.

**Fig. 1:**
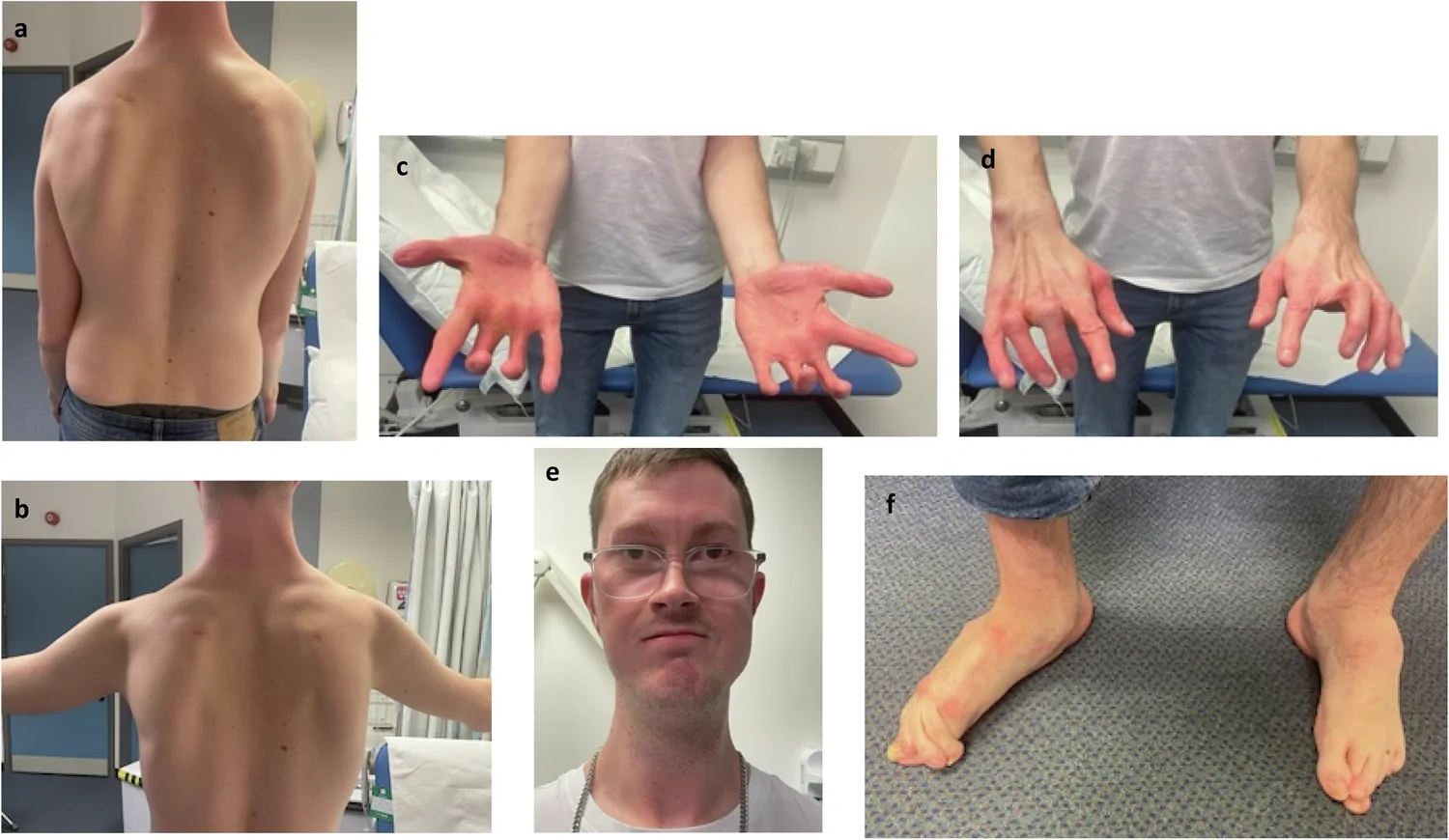
Dysmorphic and skeletal features: **a** and **b**; prominent scapulae **c** and **d**; finger contractures **e**; long, thin face and neck with low-set ears, right strabismus **f**; overlapping toes

Examination at age 26 revealed a long, thin face with a high arched palate and right strabismus. There is kyphosis, prominent scapulae, arthrogryposis (most prominent distally) and overlapping toes (See Fig. 1). Distal limb wasting in the legs more than in the upper limbs. Limited proximal upper limb range of movement, but strength is generally preserved. Normal tone in the upper limbs and lower limb spasticity. Hyper-reflexia in all four limbs.

Pertinent investigations demonstrated a normal CK. MRI C-spine showed kyphosis with narrowing of the spinal canal at C2-5, impingement of the cord, but no compression. The MRI of the brain was normal. Nerve conduction studies showed mild left ulnar neuropathy and EMG showed an excess of complex polyphasic motor units and mild myopathic motor units in proximal lower limbs. Ophthalmological exam was impacted by nystagmus but showed visual acuity R 6/18, L 6/12 with glasses, normal retina, maculae and optic discs. Colour vision was reduced 10/15 bilaterally. Flash VEPs (Visual Evoked Potentials) were notable for an unusual waveform dominated by an early positivity from both eyes, but the normal pattern reversal VEPs were felt to largely exclude significant optic nerve dysfunction, and his reduced visual acuity remained unexplained.

He was prescribed perampanel 4 mg OD for his exaggerated startle response with significant and sustained (more than one year) subjective benefit.

### Genetic results

The proband is compound heterozygous for the novel NM_001024845.3:c.1693del p.(Asp565fs) and NM_001024845.3:c.1168_1170del (p.Phe390del) variants in the *SLC6A9* gene. Parental analysis confirmed the variants to be present in *trans*.

## Discussion

GlyT1 encephalopathy due to bi-allelic *SLC6A9* gene variants is a very rare form of glycine encephalopathy and its phenotypic spectrum is incompletely delineated. The male adult we describe is, to our knowledge, the first Caucasian and the first from a non-consanguineous family. He has many clinical features that are consistent with the described phenotype. Namely, early neonatal onset with neonatal hypotonia and later hypertonicity, respiratory failure, exaggerated startle and arthrogryposis. Perampanel had a significant benefit for the exaggerated startle, possibly by modulation of excitatory/inhibitory balance via AMPA receptor antagonism, which could provide insights into treatment options for others.

He additionally demonstrates some rare or novel phenotypic features e.g. pattern of burst suppression on EEG seen in only one other case (Table 1). His significant visual impairment is unusual. GlyT1 is essential for glycinergic neurotransmission in the retinal amacrine cells, which help to process visual information. GlyT1 deficiency has been shown to impact retinal processing in both mice and humans, but exactly how this could correlate to his visual loss remains speculative and to be explored [7].

He is also, as far as we are aware, the eldest surviving person with GlyT1 encephalopathy. It is notable that after a difficult neonatal period and early childhood, his disease course seemed to improve to some degree in later childhood and has remained relatively stable since. This offers prognostic insights for future affected individuals.

Glycine is an important neurotransmitter. After its pre-synaptic release, it must be rapidly cleared from the synaptic cleft and glycine transporter 1 (GlyT1) plays an essential role in this. It is difficult to know at this stage if the prognosis will be variant-specific. Previous reported variants have included premature truncations or a frameshift variant predicted to result in premature truncation, as well as missense variants. Premature truncations were shown to result in complete loss of function in vitro [4]. Although mis-sense variants still allowed protein expression at the cell membrane, transporter activity was significantly reduced. The in-frame deletion p.(Phe390del) is located in an intracellular linker between transmembrane helix 6 and 7. No other reported variants are in the same protein region, nor is there significant knowledge of the function of this linker. It could be speculated that it may be less critical and allow more residual transporter function. From a clinical perspective, however, both types of variants in homozygous form have resulted in premature death, although it could be postulated that the combination of variants in the individual described here did retain sufficient GlyT1 function that mortality was avoided.

None of the authors declared Financial and Non-Financial Relationships and Activities and Conflicts of Interest regarding this manuscript.

## Supplementary information

Supplemental Table ACMG Criteria
