# Supplementary material for: Novel SLC6A9 variants in an adult presenting with GlyT1 encephalopathy
Source: J Hum Genet. 2026 Sep 11;71(11):761–5. doi: 10.1038/s10038-026-01497-4 (PMC13695571; doi:10.1038/s10038-026-01497-4)
Supplement: Supplementary file 1 [file 10038_2026_1497_MOESM1_ESM.pdf]

Variant details

Gene	Zygosity	Inheritance	HGVS description	Location (GRCh38/hg38)	Classification*
SLC6A9	Heterozygous		NM_001024845.3:c.1693del p.(Asp565fs)	Chr1(GRCh38):g.43997872del	Likely pathogenic

Gene-Disease Association	Glycine encephalopathy with normal serum glycine	
Inheritance	Autosomal Recessive	
**Evidence for variant classification using ACMG/AMP guidelines**:		**Evidence points^**
PM2	At very low levels in gnomAD V4.1.0 (5 heterozygotes and no homozygotes observed).	2
PVS1_mod	A frameshift variant in the penultimate exon of the gene which is predicted to escape nonsense mediated decay, and affects approximately 10% of the protein.	2
PP4_sup	The patient phenotype has quite a rare combination of clinical features, which are characteristic of SLC6A-associated disorder and for which there are a limited number of known genetic aetiologies.	1
PM3_sup	Variant in trans with a previously classified as VUS.	1
		Total: 6

Gene	Zygosity	Inheritance	HGVS description	Location (GRCh38/hg38)	Classification*
SLC6A9	Heterozygous		NM_001024845.3: c.1168_1170del (p.Phe390del)	chr1(GRCh38):g.44001428_44001430del	Likely pathogenic

Gene-Disease Association	Glycine encephalopathy with normal serum glycine	
Inheritance	Autosomal Recessive	
**Evidence for variant classification using ACMG/AMP guidelines**:		**Evidence points^**
PM2	At very low levels in gnomAD V4.1.0 (3 heterozygotes and no homozygotes observed).	2
PM4_Supporting	This variant removes a single amino acid from a moderately conserved region of the encoded protein.	2
PP4_sup	The patient phenotype has quite a rare combination of clinical features, which are characteristic of SLC6A-associated disorder and for which there are a limited number of known genetic aetiologies.	1
PM3_sup	Variant in trans with a previously classified as VUS.	1
		Total: 6

^Evidence point ranges: VUS: 0-5 (10-90% posterior probability pathogenicity); Likely pathogenic: 6-9 (90-99% posterior probability); Pathogenic: ≥10 (>99% posterior probability). Points awarded per evidence weighting supp (supporting) = 1, mod (moderate) = 2, str (strong) = 4, vstr (very strong) = 8 (ACGS 2024 variant guidelines https://www.acgs.uk.com/media/12533/uk-practice-guidelines-for-variant-classification-v12-2024.pdf).
